# *CKMT2* Promotes Breast Muscle Growth in Qiangying Ducks via Enhancing Myoblast Proliferation and Differentiation

**DOI:** 10.3390/ani15243516

**Published:** 2025-12-05

**Authors:** Longfei Xie, Dongsheng Wu, Wanli Yang, Ya Li, Jie Zhang, Zhaoyu Geng

**Affiliations:** 1 College of Animal Science and Technology, Anhui Agricultural University, Hefei 230036, China; 13188265421@163.com (L.X.); m18437969621@163.com (D.W.);; 2 Anhui Provincial Key Laboratory of Local Animal Genetic Resources Conservation and Bio-Breeding, Hefei 230036, China

**Keywords:** duck meat, breast muscle, myoblast, transcriptome, *CKMT2*, SNP

## Abstract

The duck meat industry is economically vital in animal husbandry; however, the genetic mechanisms governing skeletal muscle development and muscle yield remain incompletely understood. In this study, RNA sequencing (RNA-seq) was used to identify differentially expressed genes (DEGs) in breast muscle tissues between high and low breast muscle weight (BMW) groups of Qiangying ducks. A candidate gene, *CKMT2* (mitochondrial creatine kinase 2), was subsequently validated at the cellular and individual levels. RNA-seq analysis identified 540 DEGs, including 411 upregulated and 129 downregulated genes. GO and KEGG enrichment analyses revealed enrichment in fatty acid metabolism, fibrinolysis, and PPAR signaling pathways—processes closely linked to energy homeostasis and muscle growth. Functional validation demonstrated that *CKMT2* overexpression significantly promoted myoblast proliferation and myotube differentiation (*p* < 0.05), while knockdown exerted the opposite effects. At the genetic level, the GG genotype of *CKMT2* SNP (G.76,602,082 G>A) was associated with significantly higher BMW compared to the GA and AA genotypes (*p* < 0.05). These findings provide novel insights into the molecular basis of muscle weight variation in meat ducks and establish *CKMT2* as a key regulator of skeletal muscle growth via modulation of myoblast proliferation and differentiation. This study contributes to the theoretical foundation for improving duck muscle yield through genetic selection.

## Simple Summary

This study focuses on genetic breeding, aiming to enhance meat duck production efficiency, with findings providing theoretical and practical contributions to sustainable animal husbandry. Through transcriptome sequencing, we identified differentially expressed genes in the skeletal muscle of Qiangying ducks with varying breast muscle weights during growth and validated the functional role of *CKMT2* at both cellular and individual levels. These results advance understanding of skeletal muscle development mechanisms in ducks and establish a theoretical foundation for *CKMT2*-mediated muscle growth regulation.

## 1. Introduction

The global duck industry is a vital sector of animal agriculture, with China playing a dominant role, accounting for more than 60% of the world’s duck population and leading in both production and consumption of duck meat [1]. Although duck meat is less common than chicken or turkey in Western markets, it holds substantial cultural and economic importance across East and Southeast Asia. Improving production efficiency, particularly traits such as growth rate and breast muscle yield, has therefore become a major breeding objective.

Qiangying duck is a commercial meat-type line that has been intensively selected for rapid early growth, high feed efficiency and good viability in China. In breeding practice, Qiangying ducks are reported to reach approximately 3.35 kg body weight at 40 days of age with a feed conversion ratio of about 1.89:1 (unpublished data, Qiangying Duck Industry Group, 2023), indicating excellent potential for commercial meat production and genetic improvement.

In poultry, skeletal muscle development differs fundamentally from that in mammals, as it occurs independently of maternal uterine influence and is largely governed by genetic and incubation factors [2]. Muscle mass depends on the number, cross-sectional area, and length of individual fibers [3]. During embryogenesis, myoblasts proliferate and fuse to form multinucleated myotubes, establishing the muscle structure before hatching [4]. After hatching, further growth is mainly achieved through hypertrophy, that is, enlargement of existing fibers rather than an increase in fiber number.

Beyond genetics, environmental and nutritional conditions strongly affect muscle development and overall production outcomes in poultry and other livestock species [5]. Dietary nutrient composition, energy balance, and feed quality are known to influence growth rate, bone and muscle morphology, and metabolic efficiency. Studies in pigs and chickens have demonstrated that variations in feed ingredients or nutrient supplementation can markedly alter physiological development and tissue characteristics [6,7,8]. Therefore, appropriate dietary formulation and standardized management are prerequisites for accurately assessing genetic effects on muscle growth in meat ducks.

Skeletal muscle growth and regeneration are primarily mediated by satellite cells, which act as muscle stem cells, donate nuclei to fibers, and determine regenerative capacity [9,10]. These cells originate from progenitors expressing the transcription factors Pax3 and Pax7, which maintain the progenitor cell pool and regulate both hypertrophy and the formation of new fibers [11,12]. Muscle development is further modulated by a balance of positive and negative regulators. For example, Myostatin (MSTN) acts as a potent inhibitor of myoblast proliferation and muscle growth [13], whereas myogenic regulatory factors (MRFs) such as MyoD and Myf5 promote myogenic commitment and differentiation [14]. Together, these genes orchestrate the transcriptional network underlying muscle formation and maintenance in poultry.

The yield and quality of duck breast meat are thus determined by the complex interactions among Pax3, Pax7, MSTN, and MRFs. This genetic framework provides the foundation for improving duck meat production through both conventional selection and modern molecular approaches. Although the roles of MyoD, Myf5, and Myogenin in myoblast differentiation have been established in mammals and chickens [15], the molecular mechanisms governing muscle development in ducks remain poorly understood, particularly those linked to energy metabolism and the transition from myoblast proliferation to myotube formation.

Creatine kinase mitochondrial 2 (*CKMT2*) is the mitochondrial isoform of creatine kinase that catalyzes the reversible transfer of high-energy phosphate between ATP and creatine, thereby sustaining ATP buffering within mitochondria and at sites of contraction. *CKMT2* is anchored to the inner mitochondrial membrane and is highly expressed in oxidative tissues such as heart, brain, and skeletal muscle, where tissue-specific expression of mitochondrial creatine kinases is crucial for maintaining adenine nucleotide homeostasis and mitochondrial function [16,17]. Proteomic and transcriptomic analyses in livestock have identified *CKMT2* as a central node in muscle energy metabolism during development in swine and chickens [18,19]. At the sarcomeric M-line, *CKMT2* homodimers participate in the phosphocreatine shuttle at ATP utilization sites during muscle contraction [20], and rapid postnatal upregulation of *CKMT2* expression in extraocular muscles underscores its importance in high-energy-demanding fibers [21]. However, whether *CKMT2* directly modulates the proliferation and differentiation of avian myoblasts, and how its genetic variation influences muscle growth in meat-type ducks, remains unknown.

To address this knowledge gap, the present study employed transcriptome sequencing to identify differentially expressed genes (DEGs) associated with breast muscle weight in Qiangying ducks exhibiting high and low muscle yield. Among these, *CKMT2* was identified as a candidate gene potentially involved in myogenesis. We then combined in vitro functional assays in primary duck myoblasts with association analysis of *CKMT2* single-nucleotide polymorphisms (SNPs) and breast muscle weight at the individual level. This integrated approach aimed to elucidate the role of *CKMT2* in duck skeletal muscle development, focusing on its effects on myoblast proliferation and myotube formation, and to provide new insights for the genetic improvement of meat-type ducks.

## 2. Materials and Methods

### 2.1. Animal Samples

A total of 500 one-day-old male Qiangying ducks of similar body weight were provided by Anhui Huangshan Qiangying Duck Industry Co., Ltd. At 1 day old, ducklings with similar weights were selected and raised until 21 days old. At 21 days old, they were placed in individual feeding cages of 55 cm × 50 cm × 40 cm, respectively, with free access to drinking water and feed.

The ducks were reared in an environmentally controlled facility following standard commercial practices for meat duck production in China. The experimental diet was a nutritionally balanced commercial pellet feed, and the detailed ingredient composition is considered proprietary information by the manufacturer. However, the diet was formulated to meet the nutritional requirements for growth and development of meat ducks as specified in the national standard (GB/T 45103-2024 (China)).

Thirty healthy ducks with high body weight and another thirty with low body weight were randomly selected. At 42 days of age, approximately 5 mL of blood was collected from the wing vein of each duck into anticoagulant tubes for DNA extraction. Immediately afterwards, the ducks were euthanised by cervical dislocation, and the entire left pectoralis major muscle was dissected and weighed using an electronic balance. This value was recorded as breast muscle weight (BMW). Breast muscle samples were collected from the left pectoralis major muscle at the midpoint of the sternum, avoiding tendons and adipose tissue. This location was chosen for consistency, as it represents the central region of the pectoralis major. The top 20% of individuals with the highest breast muscle weight were defined as the high breast muscle weight group, while the bottom 20% were defined as the low breast muscle weight group.

Based on the distribution of BMW in this population, ducks in the highest 20% (n = 12) and lowest 20% (n = 12) were defined as the high-BMW and low-BMW groups, respectively. This percentile-based grouping was chosen to maximize the phenotypic contrast between groups while keeping a sufficient number of individuals for RNA-seq and histological analyses.

The left breast muscle tissue was collected from three ducks in each group. One portion of tissues was fixed in paraformaldehyde for tissue section preparation, while the other portion was quickly frozen in liquid nitrogen for RNA extraction and transcriptome sequencing.

### 2.2. Preparation of Breast Muscle Paraffin Sections and HE Staining

Fresh tissues were fixed in 4% paraformaldehyde for 24 h, trimmed, dehydrated with graded alcohol, cleared with xylene, and embedded in paraffin using a Leica EG1150H Embedding System (Leica Microsystems, Wetzlar, Germany). The paraffin blocks were sectioned to 4 μm thickness, flattened in warm water, and then baked onto glass slides. For HE staining, sections were deparaffinized, rehydrated, and stained with Harris hematoxylin and eosin. After dehydration and clearing with xylene, the slides were mounted with neutral balsam. Muscle fiber cross-sectional area and diameter were measured and analyzed using the CellSens imaging software (version 3.1.1, Olympus Corporation, Tokyo, Japan).

### 2.3. RNA Isolation and RNA-Seq Analysis

Total RNA from duck breast muscle was extracted using the Trizol reagent (Invitrogen, Carlsbad, CA, USA) according to the manufacturer’s instructions. A 1 μL RNA sample was used to measure the OD260/280 ratio using a NanoDrop™ spectrophotometer (Thermo Fisher Scientific, Waltham, MA, USA). RNA integrity and quality were assessed by 1.2% agarose gel electrophoresis. cDNA was synthesized using Hifair^®^ II 1st Strand cDNA Synthesis Kit (Yeasen Biotechnology, Shanghai, China). Transcriptome sequencing libraries were constructed and sequenced by OE Biotech Co., Ltd. (Shanghai, China) on the Illumina NovaSeq 6000 platform (Illumina, San Diego, CA, USA).

For RNA-seq, three biological replicates per group (high-BMW and low-BMW) were selected from individuals whose BMW values were closest to the respective group means. Although the sample size is relatively modest, it is within the range commonly used in livestock transcriptomic studies, and the reliability of the RNA-seq data was further supported by qRT-PCR validation of representative DEGs.

The raw reads from the transcriptome sequencing were filtered to remove reads containing adapter sequences or poly-N, reads with over 10% unknown nucleotides, and reads with more than 50% low-quality bases (Q-value ≤ 10). High-quality clean reads were obtained, and their Q30, GC content, and sequence duplication levels were calculated. The clean reads were then mapped to the Anas platyrhynchos genome (NCBI Annotation Release 104, GCF_003850225.1). The clean reads were processed by Trimmomatic v0.39 for adapter trimming and quality control (discarding reads with >10% N bases or >50% Q ≤ 10 bases), then aligned to the Anas platyrhynchos genome (GCF_003850225.1) using HISAT2 v2.2.1. Gene-level raw counts were quantified with featureCounts v2.0.1 (parameters: -t exon -g gene_id -s 2). FPKM values were calculated from these counts. DEGs were identified from raw counts using DESeq2 v1.34.0, applying thresholds of |log_2_FC| ≥ 1 and Benjamini–Hochberg FDR < 0.01.

### 2.4. Functional Analysis of Differentially Expressed Genes

Gene ontology (GO) and Kyoto Encyclopedia of Genes and Genomes (KEGG) databases were used for functional annotation and significant enrichment analysis of the identified DEGs using the clusterProfiler R package (v4.6.2). The GO database categorizes and analyzes gene functions through three main branches: biological process, molecular function, and cellular component. The KEGG database, using hypergeometric tests implemented in clusterProfiler v4.6.2, identifies biologically significant pathways that are enriched within the genomic.

### 2.5. Vector Construction

The overexpression vector pcDNA3.1-CKMT2 (oe CKMT2) and its empty vector (oe NC), along with the interference vector PGPU6-GFP-Neo-shCKMT2 (shCKMT2) and its empty vector (shNC), were constructed by (Gima Pharmaceutical Technology Co., Ltd., Shanghai, China) The constructed vectors were introduced into competent cells for amplification. The RNA oligonucleotide sequences are listed in Table 1.

To amplify the plasmids, 200 μL of an overnight bacterial culture was inoculated into 50 mL of LB medium supplemented with the appropriate selection antibiotic and incubated at 37 °C with shaking at 150 rpm overnight. The following day, the culture was centrifuged at 3000 rpm for 20 min, and plasmids were extracted using the Tiangen endotoxin-free plasmid extraction kit (DP117). Concentration and purity were measured with a NanoDrop™ spectrophotometer (Thermo Fisher Scientific, USA).

### 2.6. Isolation, Culture, and Transfection of Primary Duck Myoblasts

Primary duck myoblasts were isolated from 21-day-old embryonic duck breast muscle tissue as previously described [22]. Briefly, the tissue was minced and digested with a mixture of type I and type II collagenase (Yisheng Biotechnology, Shanghai, China) in a 1:1 ratio, and incubated in a 37 °C shaking incubator for 30 min. The cell suspension was filtered through 50-μm and 200-μm mesh filters and centrifuged at 1000 rpm for 10 min to remove the supernatant. Red blood cell lysis buffer (Beyotime Biotechnology, Shanghai, China) was added to the pellet, mixed by gentle pipetting, and allowed to stand for 15 min before being centrifuged again to remove the supernatant. The cells were washed twice and transferred to a 10 cm culture dish and cultured in complete medium (serum + DMEM, 1:9 ratio) under conditions of 37 °C and 5% CO_2_.

Cell transfection was prepared when the cell confluence reached over 65% (Lipofectamine 3000 Thermo Fisher Scientific, Shanghai, China) was used as the transfection reagent.

### 2.7. CCK8 Assay

The duck myoblasts were seeded in 96-well plates at approximately 2 × 10^3^ cells per well and cultured until ~40% confluence. Cells were then transfected with pcDNA3.1-CKMT2 (oe CKMT2), shCKMT2 or the corresponding control vectors for 24 h at 37 °C in a 5% CO_2_ incubator. Subsequently, 10 μL of CCK-8 solution (Pulei, Shanghai, China) was added to each well and the plates were incubated for 2 h at 37 °C in the dark. The absorbance was measured using a microplate reader (Thermo Fisher Scientific, USA) at the appropriate wavelength according to the manufacturer’s instructions.

### 2.8. Quantitative Real-Time PCR (qRT-PCR)

To verify the reliability of the RNA-seq results, eight genes, namely *ELXF*, *GFRA4*, *APOB*, *SLCSA1*, *PLPP4*, *CKMT2*, *CDHR5*, and *IFITM5*, were randomly selected for qRT-PCR validation. GAPDH was used as an internal reference gene, and primers were designed using Primer3 (version 0.4.0, Table 1). The reaction system contained 10 μL of traSYBR Mixture (with ROX) (Yeasen Biotechnology, China), 1 μL cDNA, 1 μL each of forward and reverse primers, and H2O to a final volume of 20 μL. Each sample was set up with three technical replicates and biological replicates. The reaction conditions were as follows: pre-denaturation at 95 °C for 4 min, denaturation at 95 °C for 35 s, annealing at 60.0 °C for 45 s, and extension at 72 °C for 45 s, with a total of 40 cycles.

### 2.9. Western Blot

Cell proteins were extracted using the efficient RIPA tissue/cell rapid lysis kit (Kulaibo Technology, Hangzhou, China) and then quantified using a BCA protein assay kit (A55860, Thermo Fisher Scientific, USA). The protein samples were polyacrylamide gel electrophoresis and then transferred to PVDF. After transfer, the membrane was washed three times with TBST and blocked with 5% BSA blocking solution for 2 h. The primary antibody, Rabbit Anti-CKMT2 (1:1000, bs-3526R-Gold, Bioss Antibodies, Beijing, China), was added and incubated overnight at 4 °C. The membrane was washed three times with TBST and incubated with secondary antibody HRP-conjugated Goat Anti-Rabbit IgG (1:1000, ab205718, Abcam, Cambridge, UK) for 2 h. Afterwards, the membrane was incubated with chemiluminescent substrate for 2 min and exposed using the UVI FireReader (UVItec Ltd., Cambridge, UK).

### 2.10. SNP Typing of CKMT2

The study was conducted using a population of 60 Qiangying ducks. Genomic DNA was extracted using the TianGen DNA Extraction Kit (DP348, Tiangen Biotech, Beijing, China) and DNA concentration was measured using a Nanodrop 2000. The quality of the DNA was assessed by 1% agarose gel electrophoresis in 1× TAE buffer at 100 V for 30 min, with DNA visualization using SYBR Green under UV light. Primers for the duck *CKMT2* gene were designed based on the gene sequence from the Ensembl database (https://www.ensembl.org/index.html?redirect=no) (accessed on 1 December 2024) (Table 1) and synthesized by Sangon Biotech (Sangon Biotech, Shanghai, China).

Subsequently, 1 µL of DNA template and 7 µL of ddH_2_O were used for the PCR reaction. The reaction conditions were as follows: pre-denaturation at 95 °C for 3 min, followed by 30 cycles of denaturation at 95 °C for 30 s, annealing for 30 s, and extension at 72 °C for 5 s, with a final extension at 72 °C for 5 min. PCR products were detected by 1% agarose gel electrophoresis and sent to Sangon Biotech for Sanger sequencing. Mutation sites were analyzed using DNA Star software (Qingke Bio, Beijing, China).

### 2.11. Statistical Analysis

Gene frequency, genotype frequency, effective allele number, and polymorphism information content were calculated using Popgene software (PopGene v1.32). Population-level results were expressed as mean ± SEM. Prior to analysis, all quantitative data underwent validation for normality and homogeneity of variance (assessed via Levene’s test) using SPSS 25.0 (IBM Corp., New York, NY, USA). If both assumptions were satisfied, parametric analyses were applied: (1) Group differences were compared using ANOVA with LSD post hoc tests; (2) SNP-trait associations were evaluated by Pearson correlation coefficients. If assumptions were violated, non-parametric alternatives were employed: (1) Kruskal–Wallis test with Dunn’s post hoc correction for group comparisons; (2) Spearman’s rank correlation for associations. Statistical analyses were executed in SPSS 25.0 (IBM Corp., USA), correlation plots generated in GraphPad Prism 9.0 (GraphPad Software, USA), and significance defined at *p* < 0.05.

## 3. Results

### 3.1. Comparison Analysis of Duck Breast Muscle Weight and Muscle Fiber Area

**Figure 1:**
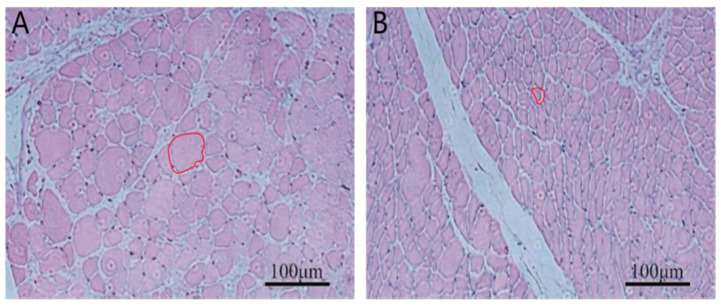
Tissue sections of breast muscle from the high and low breast muscle weight groups. (**A**): High breast muscle weight, (**B**): Low breast muscle weight. The regions are highlighted with red circles. Scale bar = 100 µm.

At 42 days of age, breast muscle weight and muscle fiber area were measured in ducks from the high- and low-BMW groups as described in the Materials and Methods. The results showed that the breast muscle weight of ducks in the high breast muscle weight group was significantly higher than that of the low breast muscle weight group (*p* < 0.01, Table 2). The muscle fiber area of ducks in the high breast muscle weight group was also significantly higher than that of the low breast muscle weight group (*p* < 0.05, Table 2, Figure 1).

### 3.2. Quality of Sequencing Data

The raw reads of RNA-seq ranged from 45.43M to 51.74M, and the Q30 values of all samples were above 93%, indicating high quality of the reads. The proportion of valid bases was greater than 99%. The average CG content of the samples was around 52%, and the base composition was balanced (Table 3). After filtering the reads, the mapping rate of the clean reads to the Anas platyrhynchos reference genome was approximately 80% (Table 3).

### 3.3. Identification of DEGs in Different Groups of Duck Breast Muscle Tissue

**Figure 2:**
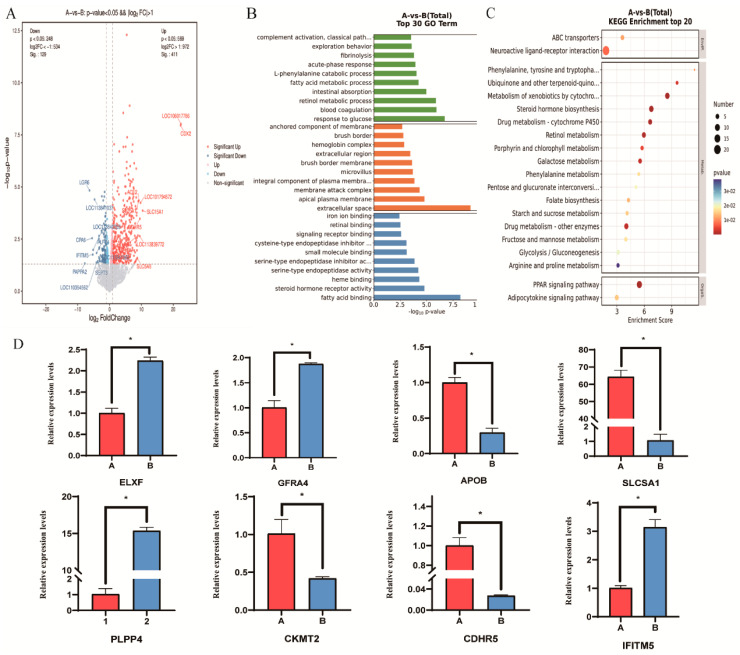
*CKMT2* coordinates proliferation-differentiation switch in avian myoblasts (**A**) Volcano plot of DEGs. (**B**) GO enrichment analysis of DEGs. (**C**) KEGG enrichment analysis of DEGs. (**D**) qPCR validation of DEGs. Note: “*” was considered a significant difference (*p* < 0.05); was considered an extremely significant difference (*p* < 0.01).

A total of 540 DEGs were identified, with 411 genes significantly upregulated and 129 genes significantly downregulated (|log_2_FC| ≥ 1, FDR < 0.01; Figure 2A). KEGG pathway enrichment analysis further revealed that these DEGs were mainly enriched in key pathways such as PPAR signaling pathway, neuroactive ligand-receptor interaction, glycolysis/gluconeogenesis, adipocytokine signaling pathway, and arginine and proline metabolism. These results suggest that amino acid metabolism and fat accumulation may be core mechanisms underlying the differences in muscle development (Figure 2B).

GO functional enrichment analysis showed that these DEGs are mainly involved in biological processes such as fatty acid metabolism, fibrinolysis, fatty acid binding, iron ion binding, steroid hormone receptor activity, membrane composition, and cysteine-type endopeptidase inhibitor activity. These results reveal that muscle growth in meat ducks is closely related to fatty acid metabolism, nutrient absorption, and fibrinolysis (Figure 2C).

### 3.4. qRT-PCR Validation of DEGs in Duck Breast Tissues

To further verify the reliability of the RNA sequencing results, the eight genes from high-throughput sequencing were randomly selected for qRT-PCR. The results showed that the expression levels of DEGs measured by qRT-PCR were consistent with the RNA-seq data, indicating the high reproducibility of the RNA-seq results in this study. In addition to *CKMT2*, the other seven DEGs (*CDHR5*, *SLC5A1*, *PLPP4*, *IFITM5*, *GFRA4*, *APOB* and *ELN*) also showed expression patterns in qRT-PCR that were consistent with the RNA-seq results, suggesting that they may also participate in the regulation of breast muscle development. Notably, we found that the expression patterns of *CKMT2* were significantly different between the high and low breast muscle weight tissues of Qiangying ducks (*p* < 0.05). *CKMT2* was enriched in pathways related to creatine kinase activity (GO:0004111), ATP binding (GO:0005524), and mitochondrial inner membrane (GO:0005743). Therefore, we speculate that *CKMT2* may play an important regulatory role in the growth and development of duck skeletal muscle.

### 3.5. CKMT2 Promotes the Differentiation of Myoblasts and the Formation of Myotubes

**Figure 3:**
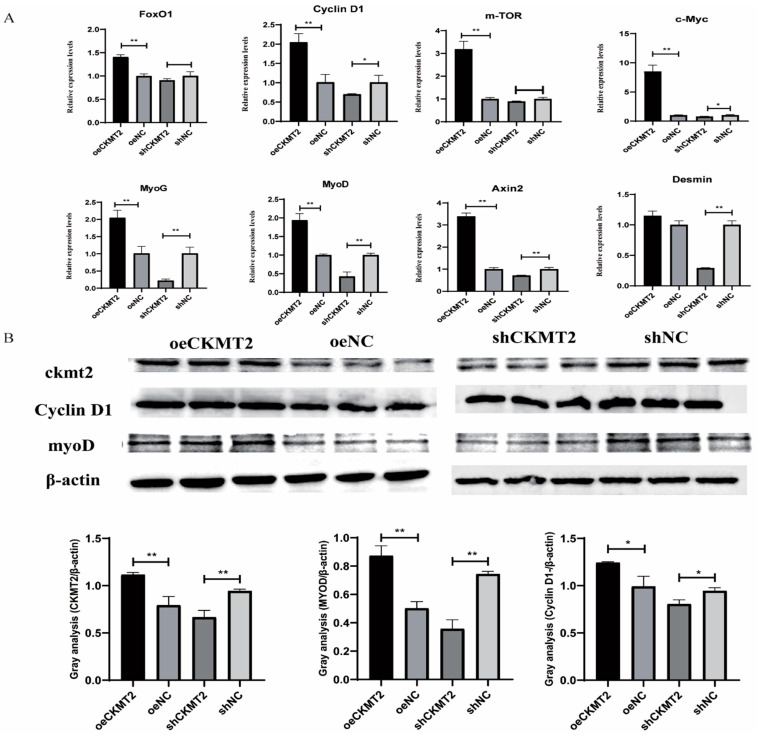
CKMT2 modulates myogenic progression via transcriptional and post-translational regulation of proliferation/differentiation markers. (**A**) Expression levels of myoblast proliferation and differentiation marker genes after overexpression and knockdown of *CKMT2*. (**B**) Protein expression levels of myoblast proliferation and differentiation marker genes after overexpression or knockdown of *CKMT2*.(** denote statistical significance by two-tailed Student’s *t*-test: * *p* < 0.05; ** *p* < 0.01.)

To explore the effect of *CKMT2* on the proliferation and differentiation of duck myoblasts, qRT-PCR was used to detect the expression of proliferation-related genes (Cyclin D1, c-Myc and mTOR) and differentiation-related genes (MyoD, MyoG and Desmin), as well as FoxO1 and Axin2, which are involved in myogenic differentiation and metabolic regulation. The results demonstrated that *CKMT2* overexpression significantly upregulated the expression levels of key regulatory genes governing myogenesis, including Cyclin D1, c-Myc and mTOR (proliferation markers), MyoD and MyoG (differentiation drivers), and FoxO1 and Axin2 (metabolic and differentiation regulators). After interfering with *CKMT2*, the expression levels of genes related to muscle differentiation were significantly reduced, but there was no significant effect on the expression of genes related to myoblast proliferation. Given that MyoD and Desmin play a positive regulatory role in myoblast differentiation, while Cyclin D1 is a key regulator of cell cycle progression and myoblast proliferation, these results suggest that *CKMT2* can promote myoblast differentiation and also exert a positive effect on myoblast proliferation. After transfection with the *CKMT2* overexpression vector in myoblasts, *CKMT2* mRNA expression was significantly increased (*p* < 0.01), while interference with *CKMT2* expression led to a significant downregulation (*p* < 0.01) (Figure 3A). Western blot analysis showed that the changes in CKMT2 protein levels were consistent with the mRNA levels (Figure 3B).

**Figure 4:**
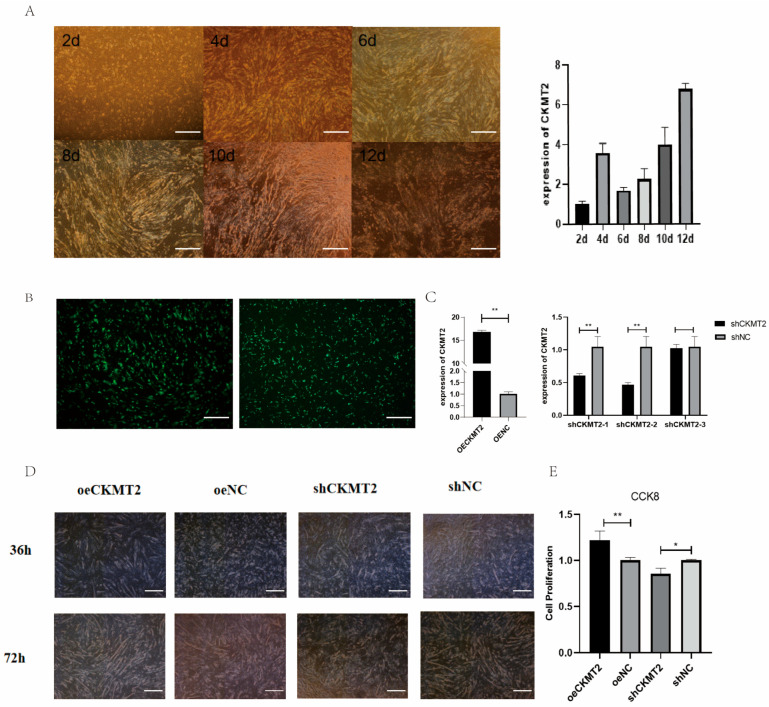
*CKMT2* modulates breast muscle development in ducks via myoblast regulation. (**A**) Growth and differentiation process of duck myoblasts. (**B**) Cell transfection with overexpression vector and shRNA. (**C**) Expression efficiency of overexpression and knockdown vectors. (**D**) Phenotypic changes in myoblasts after overexpression or knockdown of *CKMT2*. (**E**) CCK8 results. (** denote statistical significance by two-tailed Student’s *t*-test: * *p* < 0.05; ** *p* < 0.01.)

As delineated in Figure 4A, *CKMT2* expression exhibited dynamic progression during myoblast differentiation from days 2 to 12. Notably, its expression demonstrated a transient surge at day 4 (*p* < 0.05 vs. adjacent time points), coinciding with the peak phase of myogenic activation. A cumulative increase was observed over the 12-day differentiation window (*p* < 0.01, day 12 vs. day 2). Transfection efficacy was quantified 24 h post-transfection (Figure 4B). Notably, transfection with pcDNA3.1-CKMT2 (oe CKMT2) induced a 15-fold upregulation of CKMT2 mRNA compared to the empty vector control (oe NC) (*p* < 0.01). Among the three shRNA constructs (PGPU6-GFP-Neo-shCKMT2), shCKMT2-2 achieved the strongest suppression (*p* < 0.001 vs. scrambled control shNC) and was therefore selected for subsequent functional assays. Myotube formation analysis (Figure 4D) revealed that the oe CKMT2 group exhibited accelerated differentiation kinetics, with significantly increased myotube density per unit area compared with oe NC (*p* < 0.05 at 48 h; *p* < 0.01 at 72 h). In contrast, the shCKMT2-2 group showed a significant reduction in myotube numbers relative to shNC (*p* < 0.05 at 72 h). Proliferation assays (Figure 4E) further demonstrated oe CKMT2 enhanced proliferation by 38.7% compared with oe NC (*p* < 0.05). whereas shCKMT2-2 suppressed proliferation by 42.3% compared with shNC (*p* < 0.05).

### 3.6. Analysis of Single Nucleotide Polymorphisms (SNPs) in the CKMT2 Gene and Their Association with Breast Muscle Weight

Based on PCR sequencing results (Table 4), five SNPs were identified in a population of 60 ducks, with two located in non-coding regions and three in coding regions. Analysis of these SNPs revealed that all *CKMT2* loci exhibited moderate polymorphism (0.25 < *PIC* < 0.5), and all were in Hardy–Weinberg equilibrium (*p* > 0.05). In addition, G.76,602,120 and G.76,602,117 were found on the same exon, and both mutation loci showed consistent mutation frequencies (Table 5).

The breast muscle weight of Qiangying ducks with GG genotype at G.76,602,082 G. >A site (located in the exon region) was significantly higher than that of GA and AA genotypes (*p* < 0.05, Table 6). However, no significant difference in breast muscle weight was found between the GA and AA genotypes (*p* > 0.05). In addition, no significant correlation was observed for breast muscle weight at other loci.

## 4. Discussion

In meat-type ducks, slaughter performance and breast muscle yield are key determinants of economic benefit, and improving these traits is a central objective of breeding programs [23,24,25,26,27]. In the present study, Qiangying ducks with high breast muscle weight exhibited significantly greater breast muscle mass and larger muscle fiber area than those in the low breast muscle weight group, indicating that both myofiber hypertrophy and overall muscle accretion contribute to variation in carcass traits. RNA-seq analysis further revealed 540 DEGs between the two groups, reflecting substantial transcriptional remodeling of pathways related to muscle growth, metabolism, and tissue structure.

GO analysis indicated that biological processes related to cell growth, fatty acid metabolism and binding, fibrinolysis, iron ion binding, steroid hormone receptor activity, and membrane composition differ between high and low breast muscle weight ducks. These findings are consistent with previous transcriptomic studies showing that postnatal goose breast muscle hypertrophy is accompanied by increased lipid deposition and reduced leukocyte infiltration [28], that oxidative phosphorylation, ECM–receptor interaction, focal adhesion, carbon metabolism, and amino acid biosynthesis pathways are involved in skeletal muscle development in Beijing ducks [29], and that insulin and adipocytokine signaling pathways contribute to growth differences in Jinghai yellow chickens [30]. KEGG analysis in our study similarly indicated that DEGs were enriched in the PPAR signaling pathway, neuroactive ligand–receptor interaction, glycolysis/gluconeogenesis, adipocytokine signaling, and arginine and proline metabolism. PPAR family members (α, β, and γ) are important regulators of skeletal muscle lipid oxidation and metabolic flexibility [31]. Together, these results suggest that modulation of lipid and energy metabolism is a core mechanism underlying differences in breast muscle development among Qiangying ducks.

Mitochondrial oxidative metabolism represents a primary energy source during late fetal and early postnatal stages and is crucial for the onset of independent life [32]. It also has long-term effects on muscle and whole-body metabolic maturity after birth. *CKMT2*, a mitochondrial creatine kinase, transfers high-energy phosphate from mitochondria to the cytoplasmic compartment while returning ADP to the mitochondrial respiratory chain, thereby stimulating oxidative phosphorylation and supporting ATP regeneration [32,33]. In our RNA-seq data, *CKMT2* was enriched in GO terms related to creatine kinase activity, ATP binding, and the mitochondrial inner membrane, and its expression was significantly higher in breast muscle of the high breast muscle weight group than in the low group. These observations pointed to *CKMT2* as a key candidate gene linking mitochondrial energy metabolism to skeletal muscle growth in Qiangying ducks.

Functional validation in primary duck myoblasts confirmed that *CKMT2* is a positive regulator of myogenic progression. Overexpression of *CKMT2* significantly enhanced myoblast proliferation and myotube formation, whereas *CKMT2* knockdown produced the opposite effects, indicating that *CKMT2* promotes both proliferation and differentiation of duck myoblasts. This is consistent with previous reports that *CKMT2* is primarily located on the outer side of the mitochondrial inner membrane and is highly expressed in brain, heart, gastrointestinal tract, and skeletal muscle, where tissue-specific expression of mitochondrial enzymes plays a crucial role in maintaining adenine nucleotide homeostasis [16]. *CKMT2* deficiency can impair the ADP cycle, weaken mitochondrial ATP production, and lead to mitochondrial dysfunction, and the *CKMT2* gene shares high homology with multiple motifs of mitochondrial protein-coding genes, suggesting that it may influence mitochondrial biogenesis and function via regulation of related genes [17]. Moreover, proteomic studies have identified *CKMT2* as an important protein associated with energy metabolism during muscle development in swine [18], chickens [19], and other species. At the sarcomeric M-line, *CKMT2* homodimers act as structural components at ATP utilization sites and support phosphocreatine metabolism during muscle contraction [20]; in extraocular muscles, *CKMT2* expression increases rapidly after birth and remains high in adulthood, reflecting the high energy demand of these fibers [21]. Our data extend these findings by demonstrating, in an avian primary myoblast system, that *CKMT2* is not only a metabolic marker but also an active driver of the proliferation–differentiation balance that underlies breast muscle growth in meat ducks.

Beyond expression differences, we identified five SNPs within the *CKMT2* gene in Qiangying ducks, all showing moderate polymorphism and Hardy–Weinberg equilibrium. Among these, the synonymous variant G.76,602,082 G>A was significantly associated with breast muscle weight, with individuals carrying the GG genotype exhibiting higher breast muscle weight than GA and AA genotypes. The *CKMT2* gene is approximately 40 kb in length, with its translation initiation codon located about 17 kb downstream of the transcription start site, and comprises 11 exons. Its chromosomal localization varies across species, being mapped to chromosome 5q13.3 in humans and chromosome 2p16 in pigs [34,35]. In humans, mitochondrial creatine kinase exists as two interconvertible oligomeric forms, and this structural plasticity is critical for its physiological function in myocardium [36]. Studies on *CKMT2* polymorphisms have mainly focused on pigs, where several SNP loci were identified between Tibetan and Yorkshire pigs and were associated with skeletal muscle growth, muscle energy metabolism, and hypoxia resistance; differences in *CKMT2* mRNA expression between breeds further suggest that *CKMT2* positively regulates muscle formation while negatively influencing fat deposition [37]. In light of these findings, the association between G.76,602,082 G>A and breast muscle weight in Qiangying ducks implies that even synonymous mutations in *CKMT2* may affect muscle development, potentially through effects on mRNA stability, codon usage, translation efficiency, or regulatory element binding. Functional characterization of this SNP and linked variants will be required to clarify the underlying mechanism.

Although this study focused on *CKMT2* because of its strong differential expression and clear functional link to energy metabolism, several other DEGs identified in the transcriptome analysis are also involved in lipid metabolism, extracellular matrix remodeling, and signal transduction. These genes may act in concert with *CKMT2* to shape muscle growth trajectories and deserve further investigation in future work. In addition, our SNP association analysis was conducted in a single breed and with a moderate sample size, and only one growth stage was examined. Validation of *CKMT2* variants in larger and genetically diverse duck populations, as well as longitudinal studies across developmental stages, will be necessary before *CKMT2* can be confidently used as a molecular marker in breeding schemes. Nonetheless, by integrating transcriptomic profiling, cellular functional assays, and population-level association analysis, this study provides strong evidence that *CKMT2* is a key regulator of breast muscle development in Qiangying ducks and a promising target for improving meat duck performance through molecular breeding.

## 5. Conclusions

This study identified 540 DEGs between the high and low breast muscle weight groups of meat ducks through transcriptome sequencing, including 411 upregulated genes and 129 downregulated genes. Functional enrichment analysis revealed that these genes are involved in critical biological processes regulating muscle growth, such as fatty acid metabolism and the PPAR signaling pathway. Key genes, including *ACE2*, *CDHR5*, *LGR6*, *KLF5*, *NR4A1*, *CKMT2*, and *NDRG4*, were identified as potentially related to the development of breast muscle. *CKMT2* promotes myoblast proliferation. Furthermore, one SNP locus in *CKMT2* was found to be significantly associated with breast muscle weight in meat ducks, suggesting its potential as an important molecular marker for meat duck breeding. *CKMT2* enhances duck breast muscle growth via myoblast proliferation and differentiation. However, this study also has limitations, including the relatively small RNA-seq sample size, the use of only in vitro assays for *CKMT2* functional validation, and the moderate population size for SNP association analysis, which may limit the generalization of the findings. Future studies should validate the function of *CKMT2* in vivo, for example, through overexpression or gene-editing approaches in ducks, and confirm the effect of the G.76,602,082 G>A locus in larger and independent populations to fully assess the breeding value of *CKMT2* as a molecular marker.

## Figures and Tables

**Table 1:** List of qRT-PCR primers and their sequences.

Gene	Sequence (5′–3′)		Product Length/bp
*GAPDH*	FGGAGAAACCAGCCAAGTAT RCCATTGAAGTCACAGGAGA		177
*CDHR5*	FGCAGCACTGTTGCTGATTACC RTCAGGGAAATCCTTGGAGAACTTT		110
*SLC5A1*	FTTTACCGCTTGTGCTGGTCT RTCACCTGCCTCTGAAACTGAC		118
*PLPP4*	FGCAGCTCCTGGCGGG RGGATCCAAAAATTCTGTAAAG		194
*IFITM5*	FTTCTCTGTCAAGGCGCGG RGAAAAAGCCCACGGAGTCCT		192
*CKMT2*	FCATTACACTCGGCTTGCTGC RTGCAGTAGGGTAACTACCTTGAA		170
*GFRA4*	FCGCCTCTTCACCGACAACATCT RCCACGGCTGAGTTCAGGTAGGT		124
*APOB*	FGGACTGCAGGAACTGAGCAT RGATCCGGCCTTCACTTGTCA		87
*ELN*	FAGCAGCAGCAGCAGCAAAGG RCCGATGCCAGGAACACCAACAC		195
Primer name	Sequence	Annealing temperature (°C)	Product length/bp
CKMT2-12	FTTCTGTGTAGGTTGATGTTGCT RCTGCCAGGAATCACTGTTCAT	54 54.2	1044
CKMT2-34	FGGTTACTGCTCCAAGACTGAA RGGCGGTGACCACAACATT	54.2 53.4	736
CKMT2-5	FGGTTCCAGCCATCCATCCT RTCAAGCGTGTTGTGATAGAAGA	54.3 53.9	722
CKMT2-6	FACATTCCTCCTGGCTCATCC RAGGCACAAAGTGGTGAAGAAG	54.7 54.6	714
CKMT2-78	FCCTTGGCATCAGTGGAGTTC RTCTCTTGTGTCCTCTGCTCAG	54.2 55	1264
CKMT2-9	FAAGCACCAATACACTCTGACAT RCCTGGACAGACACTGGAGAG	53.8 54.7	777
Gene	Sequence		
OECKTM2	F CGACGCGTATGGCTGGCACTTTCTGTCGT R CCATCGATTCACTTCCTGCCAAACTGTGGCA		
SHCKMT2	F CACCGCCTCACTCCAGCCATTTATGTCAAGAGCATAAATGGCTGGAGTGAGGCTTTTTTG R CGGAGTGAGGTCGGTAAATACAGTTCTCGTATTTACCGACCTCACTCCGAAAAAACCTAG		

**Table 2:** Breast muscle weight and fiber area in 42-day-old ducks.

Group	Breast Muscle Weight (g)	Muscle Fiber Area (μm^2^)
High breast muscle weight	261.70 ± 4.573 ^a^	1945.7 ± 107.4 ^a^
Low breast muscle weight	180.56 ± 7.089 ^b^	1463.1 ± 95.5 ^b^

Note: Values are presented as mean ± SEM (n = 3). Different superscript letters (a, b) within the same column indicate significant differences between groups (*p* < 0.05).

**Table 3:** Summary statistics of RNA-seq results for high and low breast muscle recombination groups.

Sample	Raw Reads (M)	RawBases (G)	Clean Reads (M)	Clean Bases (G)	Valid Bases (%)	Q30 (%)	GC (%)
A1	45.43	6.81	45.40	6.77	99.41	93.03	51.45
A2	48.11	7.22	48.08	7.17	99.29	92.93	51.83
A3	49.89	7.48	49.89	7.45	99.25	93.36	50.69
B1	51.74	7.76	51.71	7.69	99.10	93.13	51.21
B2	45.79	6.87	49.53	6.81	99.16	93.47	52.15
B3	49.59	7.44	49.55	7.37	99.13	93.28	52.03

Note: A1–3 represent individuals in the high breast muscle weight group, B1–3 represent individuals in the low breast muscle weight group.

**Table 4:** Clean reads mapped to the reference genome in high and low breast muscle weight groups of ducks.

Sample	Total Reads	Total Mapped Reads	Multiple Mapped	Uniquely Mapped
A1	45,401,218	38,600,344 (85.02%)	21.80%	63.22%
A2	48,080,860	40,482,099 (84.20%)	21.78%	62.41%
A3	49,854,956	43,283,510 (86.82%)	24.89%	61.95%
B1	51,695,208	44,176,571 (85.46%)	28.35%	67.11%
B2	45,763,860	40,030,715 (87.47%)	31.35%	64.91%
B3	49,552,966	41,785,890 (84.33%)	19.56%	64.76%

**Table 5:** Genotype distribution of *CKMT2* gene mutation loci.

SNPs	Genotype Frequency	Allele Frequency	PIC	χ2	*p*
G.76,613,408 T>C	TT (0.083), TC (0.217), CC (0.700)	T (0.191), C (0.808)	0.3107	0.301	0.583
G.76,614,856 T>C	TT (0.117), TC (O.233), CC (0.650)	T (0.233), C (0.767)	0.3574	0.041	0.840
G.76,602,082 G>A	GG (0.133), GA (0.150), AA (0.716)	G (0.208), A (0.792)	0.3294	0.433	0.511
G.76,602,120 G>A	GG (0.816), GA (0.116), AA (0.066)	G (0.725), A (0.125)	0.458	0.187	0.677
G.76,602,117G>A	GG (0.816), GA (0.116), AA (0.066)	G (0.725), A (0.125)	0.458	0.187	0.677

**Table 6:** Correlation between SNP locus of *CKMT2* gene and pectoral muscle weight in ducks.

SNPs (Locus Position)	Genotype Frequency/n	Pectoral Muscle Weight
G.76,613,408 T>C (Exon)	TT (5) TC (14) CC (41)	175.16 ± 9.91 172.42 ± 25.27 184.62 ± 26.96
G.76,614,856 T>C (Exon)	TT (7) TC (14) CC (39)	184.2 ± 11.9 181.6 ± 15.1 178.6 ± 20.4
G.76,602,082 G>A (Intron)	GG (8) GA (9) AA (43)	216.04 ± 20.92 ^a^ 191.70 ± 14.31 ^b^ 178.65 ± 19.60 ^b^
G.76,602,120 G>A (Intron)	GG (49) GA (7) AA (4)	184.42 ± 18.7 191.26 ± 21.72 175.63 ± 11.38
G.76,602,117 G>A (Intron)	GG (49) GA (7) AA (4)	184.42 ± 18.7 191.26 ± 21.72 175.63 ± 11.38

Note: Values are presented as mean ± SEM (n = number of individuals with each genotype). Different superscript letters (a, b) within the same SNP locus indicate significant differences in breast muscle weight between genotypes (*p* < 0.05).

## Data Availability

The RNA-seq datasets generated in this study will be deposited in a public repository and made openly available upon acceptance; until then, the data are available from the corresponding author on reasonable request.

## Abbreviations

The following abbreviations are used in this manuscript:

CKMT2	Creatine Kinase, Mitochondrial 2
SNP	Single Nucleotide Polymorphism
GO	Gene Ontology
KEGG	Kyoto Encyclopedia of Genes and Genomes
DEGs	Differentially Expressed Genes
FDR	False Discovery Rate
FPKM	Fragments Per Kilobase per Million mapped reads
qRT-PCR	Quantitative Real-Time Polymerase Chain Reaction
CCK8	Cell Counting Kit-8

## References

[1] Gariglio M., Dabbou S., Gai F., Trocino A., Xiccato G., Holodova M., Gresakova L., Nery J., Oddon S.B., Biasato I. (2021). Black soldier fly larva in Muscovy duck diets: Effects on duck growth, carcass property, and meat quality. Poult. Sci..

[2] Albrecht E., Teuscher F., Ender K., Wegner J. (2006). Growth-and breed-related changes of muscle bundle structure in cattle. J. Anim. Sci..

[3] Joo S.T., Kim G.D., Hwang Y.H., Ryu Y.C. (2013). Control of fresh meat quality through manipulation of muscle fiber characteristics. Meat Sci..

[4] Ren L., Liu A., Wang Q., Wang H., Dong D., Liu L. (2021). Transcriptome analysis of embryonic muscle development in Chengkou Mountain Chicken. BMC Genom..

[5] Shi H., He Y., Li X., Du Y., Zhao J., Ge C. (2022). Regulation of Non-Coding RNA in the Growth and Development of Skeletal Muscle in Domestic Chickens. Genes.

[6] Muszyński S., Dajnowska A., Arciszewski M.B., Rudyk H., Śliwa J., Krakowiak D., Piech M., Nowakowicz-Dębek B., Czech A. (2023). Effect of fermented rapeseed meal in feeds for growing piglets on bone morphological traits, mechanical properties, and bone metabolism. Animals.

[7] Wojciechowska-Puchałka J., Calik J., Krawczyk J., Obrzut J., Tomaszewska E., Muszyński S., Wojtysiak D. (2024). The effect of caponization on tibia bone histomorphometric properties of crossbred roosters. Sci. Rep..

[8] Prakatur I., Miskulin M., Pavic M., Marjanovic K., Blazicevic V., Miskulin I., Domacinovic M. (2019). Intestinal morphology in broiler chickens supplemented with propolis and bee pollen. Animals.

[9] Morgan J.E., Partridge T.A. (2003). Muscle satellite cells. Int. J. Biochem. Cell Biol..

[10] Bischoff R., Heintz C. (1994). Enhancement of skeletal muscle regeneration. Dev. Dyn. Off. Publ. Am. Assoc. Anat..

[11] Ordahl C.P., Le Douarin N.M. (1992). Two myogenic lineages within the developing somite. Development.

[12] Lewandowski D., Dubińska-Magiera M., Migocka-Patrzałek M., Niedbalska-Tarnowska J., Haczkiewicz-Leśniak K., Dzięgiel P., Daczewska M. (2020). Everybody wants to move-Evolutionary implications of trunk muscle differentiation in vertebrate species. Semin. Cell Dev. Biol..

[13] Mcpherron A.C., Lawler A.M., Lee S.J. (1997). Regulation of skeletal muscle mass in mice by a new TGF-beta superfamily member. Nature.

[14] Shirakawa T., Toyono T., Inoue A., Matsubara T., Kawamoto T., Kokabu S. (2022). Factors Regulating or Regulated by Myogenic Regulatory Factors in Skeletal Muscle Stem Cells. Cells.

[15] Buckingham M. (2001). Skeletal muscle formation in vertebrates. Curr. Opin. Genet. Dev..

[16] Ehrlich K.C., Deng H.W., Ehrlich M. (2021). Epigenetics of Mitochondria-Associated Genes in Striated Muscle. Epigenomes.

[17] Klein S.C., Haas R.C., Perryman M.B., Billadello J., Strauss A. (1991). Regulatory element analysis and structural characterization of the human sarcomeric mitochondrial creatine kinase gene. J. Biol. Chem..

[18] Voillet V., San Cristobal M., Père M.C., Billon Y., Canario L., Liaubet L., Lefaucheur L. (2018). Integrated Analysis of Proteomic and Transcriptomic Data Highlights Late Fetal Muscle Maturation Process. Mol. Cell. Proteom. MCP.

[19] Bottje W., Kong B.-W., Reverter A., Waardenberg A.J., Lassiter K., Hudson N.J. (2017). Progesterone signalling in broiler skeletal muscle is associated with divergent feed efficiency. BMC Syst. Biol..

[20] Porter J.D., Merriam A.P., Gong B., Kasturi S., Zhou X., Hauser K.F., Andrade F.H., Cheng G. (2003). Postnatal suppression of myomesin, muscle creatine kinase and the M-line in rat extraocular muscle. J. Exp. Biol..

[21] Cheng G., Porter J.D. (2002). Transcriptional profile of rat extraocular muscle by serial analysis of gene expression. Investig. Ophthalmol. Vis. Sci..

[22] Hu X., Liu Y., Tang B., Hu J., He H., Liu H., Li L., Hu S., Wang J. (2024). Comparative transcriptomic analysis revealed potential mechanisms regulating the hypertrophy of goose pectoral muscles. Poult. Sci..

[23] Hu Z., Cao J., Ge L., Zhang J., Zhang H., Liu X. (2021). Characterization and Comparative Transcriptomic Analysis of Skeletal Muscle in Pekin Duck at Different Growth Stages Using RNA-Seq. Animals.

[24] Wu P., Dai G., Chen F., Chen L., Zhang T., Xie K., Wang J., Zhang G. (2018). Transcriptome profile analysis of leg muscle tissues between slow- and fast-growing chickens. PLoS ONE.

[25] Christofides A., Konstantinidou E., Jani C., Boussiotis V.A. (2021). The role of peroxisome proliferator-activated receptors (PPAR) in immune responses. Metab. Clin. Exp..

[26] Picard B., Lefaucheur L., Berri C., Duclos M.J. (2002). Muscle fibre ontogenesis in farm animal species. Reprod. Nutr. Dev..

[27] Forsey K.E., Ellis P.J., Sargent C.A., Sturmey R.G., Leese H.J. (2013). Expression and localization of creatine kinase in the preimplantation embryo. Mol. Reprod. Dev..

[28] Huang J., Xiong X., Zhang W., Chen X., Wei Y., Li H., Xie J., Wei Q., Zhou Q. (2024). Integrating miRNA and full-length transcriptome profiling to elucidate the mechanism of muscle growth in Muscovy ducks reveals key roles for miR-301a-3p/ANKRD1. BMC Genom..

[29] Wallimann T., Tokarska-Schlattner M., Schlattner U. (2011). The creatine kinase system and pleiotropic effects of creatine. Amino Acids.

[30] Wu N., Gu T., Lu L., Cao Z., Song Q., Wang Z., Zhang Y., Chang G., Xu Q., Chen G. (2019). Roles of miRNA-1 and miRNA-133 in the proliferation and differentiation of myoblasts in duck skeletal muscle. J. Cell. Physiol..

[31] Eratalar S.A., Okur N., Yaman A. (2022). The effects of stocking density on slaughter performance and some meat quality parameters of Pekin ducks. Arch. Anim. Breed..

[32] Rao S., Morales A.A., Pearse D.D. (2015). The Comparative Utility of Viromer RED and Lipofectamine for Transient Gene Introduction into Glial Cells. BioMed Res. Int..

[33] Gao W., Cao Z., Zhang Y., Zhang Y., Zhao W., Chen G., Li B., Xu Q. (2023). Comparison of carcass traits and nutritional profile intwo different broiler-type duck lines. Anim. Sci. J..

[34] Davoli R., Fontanesi L., Zambonelli P., Bigi D., Gellin J., Yerle M., Milc J., Braglia S., Cenci V., Cagnazzo M. (2002). Isolation of porcine expressed sequence tags for the construction of a first genomic transcript map of the skeletal muscle in pig. Anim. Genet..

[35] Richard I., Devaud C., Cherif D., Cohen D., Beckmann J.S. (1993). The gene for creatine kinase, mitochondrial 2 (sarcomeric; CKMT2), maps to chromosome 5q13.3. Genomics.

[36] Walterscheid-Müller U., Braun S., Salvenmoser W., Meffert G., Dapunt O., Gnaiger E., Zierz S., Margreiter R., Wyss M. (1997). Purification and characterization of human sarcomeric mitochondrial creatine kinase. J. Mol. Cell. Cardiol..

[37] Wang Z., Duan M., Lu F., Wang S., Qiang J., Tan Z., Zhang J., Shang P. (2020). Polymorphism and Tissue Expression Patterns of CKMT2 Gene in Tibetan and Yorkshire Pigs. China Anim. Husb. Vet. Med..

